# Bile Collected From the Normal Gallbladder of Patients During Surgery Has Simple Bacterial Flora

**DOI:** 10.7759/cureus.25681

**Published:** 2022-06-06

**Authors:** Shin Watanabe, Masaaki Minagawa, Tadashi Shinoda, Daisuke Motooka, Mari Tohya, Teruo Kirikae, Shota Nakamura, Akio Saiura

**Affiliations:** 1 Department of Microbiome Research, Juntendo University Graduate School of Medicine, Tokyo, JPN; 2 Department of Hepatobiliary-Pancreatic Surgery, Juntendo University Graduate School of Medicine, Tokyo, JPN; 3 Department of Corporate Strategy, Asahi Quality & Innovations Ltd., Moriya, JPN; 4 Department of Infection Metagenomics, Genome Information Research Center, Osaka University Research Institute for Microbial Diseases, Suita, JPN

**Keywords:** human, rrna genes, microbiome, bile, gallbladder

## Abstract

Background

Bile inhibits bacterial growth because it is rich in bacteriostatic compounds such as bile acids. Analytical techniques using a high-intensity sequencer recently revealed bacterial flora in the bile of normal gallbladders in brain-dead patients. Therefore, we performed a microbial flora analysis of bile collected from pathologically normal gallbladders surgically removed from patients with hepatobiliary pancreatic diseases and normal liver function.

Methods

Bacterial DNA was extracted from bile samples and analyzed using 16S rRNA sequencing.

Results

The culture results of all 12 bile samples were negative. However, the results of the 16S ribosome gene analysis suggested the presence of bacterial flora in all samples. The phyla *Firmicutes*, *Proteobacteria*, *Actinobacteria*, and, more specifically, the genera *Anaerobacillus*, *Delftia*,* Bacillus*, *Ralstonia*, *Ochrobactrum*, *Acidovorax*, and *Curvibacter *were detected in all 12 samples. The results of the 16S rRNA gene profile analysis revealed that *Anaerobacillus* and *Delftia* accounted for 58.62%-87.63% of the bacteria identified in each sample.

Conclusion

In a bacterial flora analysis targeting the 16S ribosomal gene, a specific bacterial flora was detected in bile collected from the pathologically normal gallbladders of patients with hepatobiliary pancreatic diseases. Although a diverse bacterial flora was previously reported in the bile of brain-dead patients, the present results revealed a simple bacterial flora with no diversity in the bile samples.

## Introduction

Newly developed, high-efficient gene analysis technology has contributed to advances in bacterial microbiome research targeting intestinal bacteria, its relationship with various diseases, and their etiologies [1-2]. Difficulties are associated with examining the functional role of bacteria using a 16S rRNA gene analysis alone; however, this new technology facilitates the identification of bacterial species that are challenging to detect using normal culture techniques, as they require only a small bacterial sample.

The bacterial flora within an organ has been implicated in the pathology of disease [3-4]. Bile in the gallbladder was previously considered to be sterile in healthy patients because it does not provide a suitable environment for the survival and growth of bacteria [5]. However, a recent study reported a diverse bacterial flora in the gallbladder bile of seemingly healthy individuals [6]. The presence of a small amount of a unique bacterial flora in bile that is difficult to culture with normal methods has been suggested to inhibit the invasion of other pathogenic bacteria [6].

Genetic analysis of healthy porcine gallbladder bile recently revealed specific bacteria in gallbladder bile [7]. Age-related changes in the bile microbiome of rabbits have also been reported [8]. However, the bile of healthy humans cannot be sampled for ethical reasons. Due to the high detection sensitivity of genetic analyses, DNA sample contamination always exists. Furthermore, the test procedure may be a risk factor for biliary tract infection, even when a bacterial flora analysis is conducted via endoscopic retrograde cholangiopancreatography (ERCP), during which bile collection is possible [9-11]. Until recently, the bacterial flora of bile in healthy gallbladders had not been analyzed for ethical reasons. Molinero et al. aseptically collected and examined bile from the gallbladders of brain-dead patients with abnormalities in the biliary system and detected bacteria at the genetic level [12]. However, since these patients were likely to have had an underlying disease, the continual presence of specific bacteria in the normal gallbladder of healthy individuals cannot be assumed. Therefore, in the present study, bile was collected from patients with a very low risk of biliary tract infection and subjected to a 16S ribosomal gene analysis to confirm the presence of a microbiome.

This article was previously posted to the Research Square preprint server on September 15, 2021.

## Materials and methods

Patients

Bile samples were collected from the gallbladders of 12 patients (nine men and three women; age range, 43-89 years) who underwent hepatobiliary and pancreatic surgeries for malignant or non-malignant causes between August 2018 and May 2019 at Juntendo University Hospital (Tokyo, Japan) and required simultaneous cholecystectomy for procedural reasons. The gallbladders included in this study were macroscopically free of inflammation and infection, and bile samples were collected aseptically during surgery by a hepatobiliary and pancreatic surgeon (Table 1).

**Table 1:** Clinical characteristics and biochemical data of the study subjects WBC: white blood cell count, CRP: C-reactive protein, T-bil: total bilirubin, γ-GTP: γ-glutamyl transpeptidase, AST: aspartate aminotransferase, ALT: alanine aminotransferase, CA19-9: carbohydrate antigen 19-9

Patient No.	Sex	Age	Disease	Surgical procedure	WBC	CRP	T-bil	Γ-GTP	AST	ALT	Lipase	CA19-9
					reference ranges for blood tests							
					M: 3.9-9.7 ×10^9^/L F: 3.6-8.9 ×10^9^/L	<0.30 mg/dL	0.4-1.2 mg/dL	0-75 U/L	5-37 U/L	6-43 U/L	14-56 U/L	<37 U/mL
1	Male	70	hepatocellular carcinoma	hepatectomy of segments 5 and 6	6.3	0.08	0.63	27	39	41	61	1
2	Female	69	pancreatic neuroendocrine tumor	pancreatoduodenectomy	6.0	0.29	1.62	23	16	13	33	6
3	Female	44	solid-pseudopapillary neoplasm	pancreatoduodenectomy	7.8	0.04	0.47	13	20	15	37	5
4	Male	71	pancreatic head cancer	pancreatoduodenectomy	4.7	2.48	0.42	15	25	21	255	10
5	Male	63	pancreatic body cancer	resection of the pancreatic body and tail	4.2	0.05	0.6	20	16	15	45	1712
6	Male	48	gastrointestinal stromal tumor	partial duodenectomy	3.9	0.04	2.1	61	30	26	49	4
7	Male	84	metastatic liver cancer	hepatectomy of segments 4 and 5	5.1	0.05	0.7	13	16	13	NA	8
8	Male	70	pancreatic body cancer	resection of the pancreatic body and tail	3.7	0.17	0.58	50	35	37	121	8
9	Male	70	pancreatic neuroendocrine tumor	pancreatoduodenectomy	5.5	0.05	0.61	15	20	14	30	24
10	Female	89	hepatocellular carcinoma	enucleation of the liver	7.5	0.34	0.5	23	18	11	21	6
11	Male	43	pancreatic body cancer	pancreatoduodenectomy	5.6	0.02	1.16	30	26	43	12	19
12	Male	68	hepatocellular carcinoma	hepatic anterior segmentectomy	3.1	0.03	0.39	56	24	17	70	NA

Bile was withdrawn from the fundus of the gallbladder using an 18G needle. The specimens collected were immediately divided into 2 cc sterile testing tubes and stored at -80°C for later analyses. Bile samples were aerobically cultured with blood agar/bromothymol blue agar and anaerobically cultured with phenethyl alcohol/Bacteroides bile esculin agar medium immediately in the in-hospital laboratory. All patients received an infusion of 2 g cefmetazole one to two hours before cholecystectomy. The gallbladder was included in the analysis only for those cases in which the gallbladder was diagnosed as normal with no abnormal pathological findings by a pathologist in the hospital after surgery.

Patients who had no gallstones, a pathologically normal gallbladder, no hepatobiliary enzyme abnormalities, no inflammatory markers, and had not undergone ERCP or biliary drainage or received antibiotics three months before the present study were considered to have a healthy gallbladder and were included in analyses.

All patients provided informed consent before the procedure. The present study was approved by the Juntendo University Hospital Ethics Committee (approval number: JHS18-0309) and was conducted according to the principles of the Declaration of Helsinki.

DNA isolation

Bacterial DNA was extracted from bile samples using the DNeasy PowerSoil Kit (QIAGEN, Venlo, the Netherlands). Specimens were stored under the same conditions and were processed by trained staff using identical protocols. Samples were collected using sterile techniques for quality control. Polymerase chain reaction (PCR) reagents were regularly checked for environmental contaminants. PCR reactions had appropriate controls (without a template) to exclude DNA contaminants. To control the quality of sequencing, we used a ‘negative’ (reagent-only) control and checked for background contamination and the rate of sequencing errors.

Preparation of the 16S rRNA gene sequencing library

A library was created for each specimen using a primer set (27Fmod: 5ʹ AGR GTT TGA TCM TGG CTC AG 3ʹ and 338R: 5ʹ TGC TGC CTC CCG TAG GAG T 3ʹ) targeting the V1-V2 region of the 16S ribosomal gene based on the guidelines of the Illumina 16S Metagenomic Sequencing Library Preparation Guide [13]. MiSeq (Illumina, Inc., San Diego, CA) was used to perform a 251-base pair (bp) pair-end sequencing of this amplicon.

Sequence processing

The 251-bp paired-end sequence obtained from each specimen was processed using QIIME2 (version 2021.4; https://docs.qiime2.org/2021.4/data-resources/#silva-16s-18s-rrna) [14]. The Illumina MiSeq pair-end sequence (Fastq format) was demultiplexed (demux) to QIIME2. The DADA2 plugin was then used to trim the ends for an average quality score ≥37, and low-quality sequences and chimeric sequences were removed [15]. Therefore, the sequences obtained from each specimen had a minimum number of reads of 68,185, a maximum number of reads of 165,362, and an average number of reads of 102,623. Each sequence was then subjected to MAFFT multiple sequence alignment. Positional conservation and gap filtering were conducted using the mask plugin.

Sequence analysis

A phylogenetic tree was created using the FastTree plugin and midpoint-root plugin. Silva-138-99 sequences were obtained from the QIIME 2 website, and a dataset of sequence data spliced from the region bracketed by the 27Fmod and 338R primers and operational taxonomic units was created [13]. Class names in this dataset were extrapolated from sequence data obtained from samples as classifiers in the QIIME2 q2-feature-classifier plugin. The datasets generated during and/or analyzed during the current study are available from the corresponding author on reasonable request.

## Results

**Figure 1:**
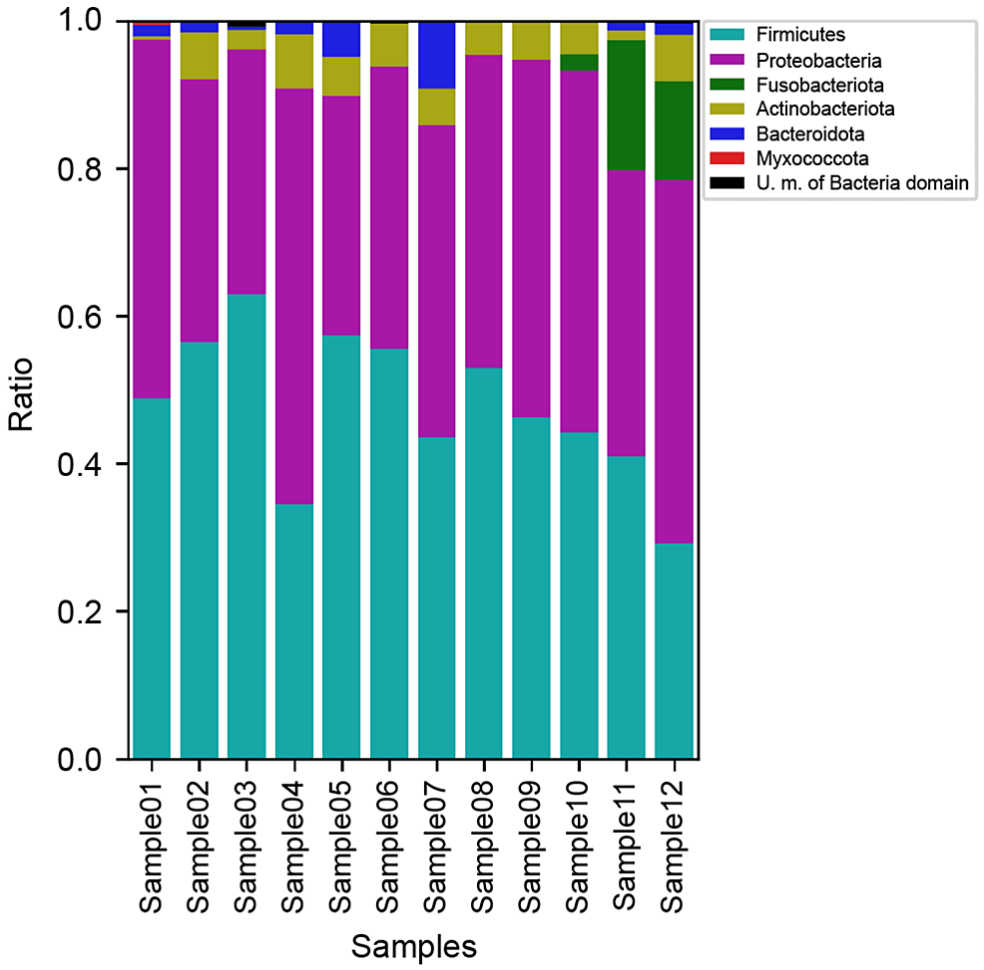
16S ribosomal gene analysis (phylum)

Bacterial growth was not detected in aerobic or anaerobic cultures of any of the 12 bile specimens collected. However, the phyla *Firmicutes*, *Proteobacteria*, and *Actinobacteria* were found in all specimens during the 16S ribosomal gene analysis and accounted for more than 80% of the flora detected. The phylum Fusobacteria was identified in 3/12 specimens (Figure 1).

**Figure 2:**
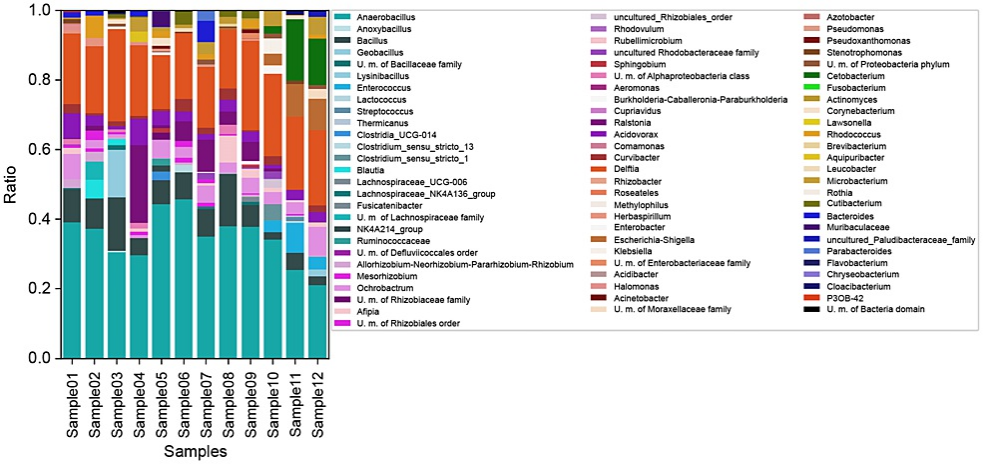
16S ribosomal gene analysis (genus)

Within the phylum *Firmicutes*, the genera *Anaerobacillus* (33.41 ± 7.0441% of the flora detected), and *Bacillus* (6.623 ± 3.8955% of the flora detected) were present in all specimens based on the 16S rRNA gene profile analysis. All bile samples contained the following genera in the phylum *Proteobacteria*: Delftia (18.68 ± 4.3935% of the flora detected), *Ralstonia* (4.14 ± 6.1401% of the flora detected), *Ochrobactrum* (4.518 ± 2.3837% of the flora detected), *Acidovorax* (3.016 ± 1.943% of the flora detected), and Curvibacter (1.561 ± 1.0242% of the flora detected). No bacteria of the phylum *Actinobacteria* were identified in any sample. Based on the 16S rRNA gene profile analysis, bacteria in the genera *Anaerobacillus* and *Delftia* accounted for 42.53-64.63% of the flora detected in each specimen (Figure 2).

## Discussion

​​​​​​The present results revealed simple bacterial flora with no diversity in gallbladder bile. The functional significance of the bacterial flora identified in this study remains unclear.

Bile acids exhibit antibacterial activities that dissolve bacterial membranes and damage bacterial DNA; therefore, bile in the biliary tract was previously considered to be sterile in healthy individuals [16]. However, some bacteria, such as *Salmonella spp.* and *Listeria monocytogenes*, may survive in the gallbladder and have been implicated in infections and the development of gallstones [17-18].

A bacterial flora analysis, based on gene sequencing using a high-efficiency sequencer, recently provided a new perspective in the field of microbiology. Flora that cannot be identified using conventional morphological cultures may be detected using gene sequencing, thereby allowing for flora classification. Bacterial genome analysis of bile in a gallbladder without inflammation, which is considered to be a low biomass environment, should be carefully evaluated with attention to the method of collection, preservation, and environment of DNA extraction of bile samples. The characteristics of the PCR kit itself always need to be considered. Recent studies reported the presence of bacteria in the gallbladder bile of relatively healthy individuals. Gene sequencing has been used to detect bacteria in the gallbladder bile of healthy swine and rabbits [7-8]. However, the bile of healthy humans cannot be sampled for ethical reasons, and the collection of bile using an endoscope is limited by contamination. Furthermore, the gallbladder of a healthy human cannot be excised for a pathological examination. To overcome these limitations, Molinero et al. performed gene sequencing on bile collected from the gallbladders of brain-dead patients and identified a bacterial flora that was not detected using conventional bacterial cultures [12]. The bacterial flora of bile in patients with cholelithiasis was also examined in that study, and the findings obtained revealed differences from that in patients with healthy gallbladders. However, the medical histories of brain-dead patients were diverse, and a pathological examination of the gallbladder and detailed liver function tests are typically not performed on these patients. In the present study, bile samples were collected from patients with normal liver function and a pathologically normal gallbladder who did not receive any antibiotics or undergo any procedures that may have affected the biliary system prior to the initiation of the study. However, most of the patients examined had cancer, which may have affected the results obtained. Although these patients had no medical history suggestive of inflammation and no bias toward a specific disease, they all had pancreaticobiliary diseases. Therefore, based on laboratory data, we considered the likelihood of infected bile in the gallbladder to be low. In the present study, a microbiome was identified in all bile samples using 16S rRNA gene sequencing.

The composition of the bacterial flora in bile in the present study was less diverse than that reported by Molinero et al. who reported that the phylum Bacteroidetes accounted for 30% of the phyla detected [12]. In the present study, the phyla *Firmicutes* and *Proteobacteria* accounted for 80% of the flora detected, the diversity of which was low.@@

At the genus level, *Anaerobacillus* and *Delftia* accounted for most of the bacteria in each specimen. Molinero et al. detected bacteria predominantly from the genera *Lactococcus*, *Propionibacterium*, *Bifidobacterium*, and *Bradyrhizobium*, which differs from the present results. These differences may be attributed to the methodologies used, with the QIIME system being employed in the previous study and the QIIME2 system in the present study. Furthermore, a previous study demonstrated that an examination of intestinal bacteria in the V1-2 region rather than in the V3-4 region is more suitable for Japanese individuals [19]. Therefore, we investigated the V1-2 region as bile is affected by the bacterial flora of the gastrointestinal tract.

The biliary tract of a healthy person was previously considered to be a sterile environment based on established culture tests. Although we collected samples from surgical patients, our findings from sequencing indicate the presence of flora specific to the gallbladder bile. This suggests that the bacteria may have entered the gallbladder in a retrograde manner from the duodenum or portal vein. When the duodenal papilla functions normally, duodenal fluid (including bacteria) does not enter the bile duct. A previous study reported that distal cholangiocarcinoma may impair the function of the duodenal papilla, leading to the accumulation of bile in the bile duct [18]; however, papillary dysfunction was not observed in the present study. Bile is constantly produced and excreted into the duodenum when food is consumed. Therefore, the presence of duodenal bacteria in gallbladder bile is only expected under pathological conditions such as biliary obstruction. As another hypothesis, if bacteria survive in bile through portal veins from the intestines, the differences observed between the present results and the findings of Molinero et al. [12] are consistent with a previous study showing a unique intestinal flora in Japanese patients, which differed from those of Chinese and Western patients [20].

The present study was performed to establish whether bacterial flora is present in bile in the normal gallbladder. Of note, the subjects were not truly healthy and, additionally, the sample size was limited by strict criteria, i.e., including only those who had not undergone ERCP with normal data of the hepatobiliary system. Furthermore, biases affecting the analysis of low biomass samples, such as variable regions set during genome analysis and different analysis software, could not be eliminated and only Japanese patients were included. These factors may have limited the results of the study.

Further studies involving functional and quantitative evaluations of the bacterial flora present in the gallbladder are warranted.

## Conclusions

The results of the 16S rRNA gene profile analysis revealed that *Anaerobacillus *and *Delftia* accounted for 58.62-87.63% of the bacteria identified in each sample of bile in the gallbladder. Our findings indicate the presence of bile-specific bacteria in the normal gallbladder. Thus, future studies are needed to fully elucidate the role of these bacteria.
